# Atheroprone flow activates inflammation via endothelial ATP-dependent P2X7-p38 signalling

**DOI:** 10.1093/cvr/cvx213

**Published:** 2017-11-06

**Authors:** Jack P Green, Celine Souilhol, Ioannis Xanthis, Laura Martinez-Campesino, Neil P Bowden, Paul C Evans, Heather L Wilson

**Affiliations:** 1 Department of Infection, Immunity & Cardiovascular Disease, University of Sheffield, Beech Hill Road, Sheffield S10 2RX, UK;; 2 Bateson Centre, University of Sheffield, Sheffield, UK;; 3 INSIGNEO Institute, University of Sheffield, Sheffield, UK

**Keywords:** P2X7, Atherosclerosis, Shear stress, ATP, Endothelium

## Abstract

**Objective:**

Atherosclerosis is a focal disease occurring at arterial sites of disturbed blood flow that generates low oscillating shear stress. Endothelial inflammatory signalling is enhanced at sites of disturbed flow via mechanisms that are incompletely understood. The influence of disturbed flow on endothelial adenosine triphosphate (ATP) receptors and downstream signalling was assessed.

**Methods and results:**

Cultured human endothelial cells were exposed to atheroprotective (high uniform) or atheroprone (low oscillatory) shear stress for 72 h prior to assessment of ATP responses. Imaging of cells loaded with a calcium-sensitive fluorescent dye revealed that atheroprone flow enhanced extracellular calcium influx in response to 300 µM 2'(3')-O-(4-Benzoylbenzoyl) adenosine-5'-triphosphate. Pre-treatment with pharmacological inhibitors demonstrated that this process required purinergic P2X7 receptors. The mechanism involved altered expression of P2X7, which was induced by atheroprone flow conditions in cultured cells. Similarly, *en face* staining of the murine aorta revealed enriched P2X7 expression at an atheroprone site. Functional studies in cultured endothelial cells showed that atheroprone flow induced p38 phosphorylation and up-regulation of E-selectin and IL-8 secretion via a P2X7-dependent mechanism. Moreover, genetic deletion of P2X7 significantly reduced E-selectin at atheroprone regions of the murine aorta.

**Conclusions:**

These findings reveal that P2X7 is regulated by shear forces leading to its accumulation at atheroprone sites that are exposed to disturbed patterns of blood flow. P2X7 promotes endothelial inflammation at atheroprone sites by transducing ATP signals into p38 activation. Thus P2X7 integrates vascular mechanical responses with purinergic signalling to promote endothelial dysfunction and may provide an attractive potential therapeutic target to prevent or reduce atherosclerosis.

## 1. Introduction

Atherosclerosis is a chronic inflammatory disease. Despite its promotion by several systematic risk factors (such as obesity, age, cholesterol, and smoking), atherosclerosis is a focal disease which occurs predominantly at distinct points of the arterial tree, such as at bends and branch points.^1^ Endothelial cells are exquisitely sensitive to shear stress, the mechanical drag imposed on the vessel wall by the blood flow. Atheroprone sites are exposed to disturbed blood flow generating low wall oscillatory shear stress. In contrast, atheroprotected regions of the vascular tree are exposed to a high magnitude and uniform wall shear stress.^2^ Shear stress regulates key endothelial processes that influence atherogenesis including inflammation and proliferation. Low shear stress is associated with enhanced expression of inflammatory molecules, including adhesion molecules and chemokines that direct the migration of leukocytes into the arterial wall, thereby driving atherosclerosis.^3^

Purinergic signalling controls multiple processes in the vasculature. Extracellular nucleotides have been documented to regulate vasodilation of blood vessels through nitric oxide production,^4–6^ indicating that they have a role in mediating atheroprotection. However, adenosine triphosphate (ATP) signalling has also been implicated with vascular dysfunction, with several studies reporting that excessive ATP-signalling induced expression of a range of chemokines and adhesion molecules, subsequently enhancing adhesion of leukocytes to the endothelium.^7–10^ This process relies predominantly on the ATP-gated receptors P2X4 and P2X7. Endothelial P2X4 and P2X7 receptor expression has been reported to be increased under inflammatory conditions *in vitro*^10^^,^^11^ and P2X7 expression is up-regulated in the atherosclerotic plaque^12^^,^^13^ contributing towards atherosclerosis development.^13^^,^^14^ A recent study demonstrated that genetic deletion of P2X7 reduced lesion formation in hypercholesterolemic mice; this was associated with a reduction in leukocyte rolling and adhesion^14^; however, the molecular mechanisms linking P2X7 to this initiation of atherogenesis and the expression and function of P2X7 at atheroprone endothelial sites was not explored.

Here we studied the effects of prolonged flow on the expression and function of P2X receptors. We demonstrate that prolonged atheroprone flow primes endothelial cells for enhanced ATP responses, attenuated CD39 ATPase activity and enhanced inflammatory activation, whereas atheroprotective flow prevented this ATP-activation pathway. The mechanism involves induction of P2X7 by atheroprone flow, leading to an elevated calcium influx response and downstream p38-dependent inflammatory activation, evidenced using *in vitro* and *in vivo* models. Our observation provides a novel mechanism for enhanced inflammation at sites of disturbed flow and suggests that therapeutic targeting of the P2X7-calcium influx-p38 pathway may prevent or treat atherosclerosis.

## 2. Methods

### 2.1 Antibodies and reagents

Specific antibodies used, were targeting: P2X7 (APR-008, Alomone); P2X4 (APR-002, Alomone); PDHX (H-130, Santa Cruz); p-p38 Thr180/Tyr182 (28B10, Cell Signalling Technologies); E-selectin (NBP1-45545, Novus Biologicals); CD31-AlexaFluor488 (Clone Mec13.3, Biolegend), and CD39-FITC (Clone A1, Biolegend). HRP-conjugated secondary antibodies were from Dako. AlexaFluor conjugated antibodies, TO-PRO-3 and aqueous mounting media (Prolong Gold Antifade Mountant) were from Invitrogen. All other reagents were from Sigma Aldrich unless specified.

### 2.2 Human umblical vein endothelial cell isolation and culture

Human umbilical vein endothelial cells (HUVECs) were isolated from umbilical cords donated by informed consent (ethical approval: Sheffield REC 10/H1308/25 according to the principles outlined in the Declaration of Helsinki) by incubating the vein with collagenase (*Clostridium histolyticum*) for 20 min and collecting the flow through. Isolated cells were cultured in gelatin [1% (w/v)] coated culture flasks in M199 media (Life Technologies) supplemented with foetal bovine serum [10% (v/v)], new-born bovine serum [10% (v/v)], 0.4 mM L-glutamine, 100 U/mL penicillin, 100 μg/mL streptomycin, 2.5 μg/mL amphotericin-B, 90 μg/mL heparin and 10 μg/mL endothelial cell growth supplement (Millipore). HUVEC were used for experiments at P3 or P4. All replicates represent separate HUVEC donors.

### 2.3 HUVEC exposure to shear stress

HUVEC were exposed to shear stress for 72 h using either the ibidi parallel plate pump system or the orbital shaker system. When using the ibidi pump system, HUVEC were grown to confluence on gelatin-coated ibidi μ-Slides I 0.4 Luer (ibidi GmbH). For atheroprotective flow, HUVEC were exposed to +4 dyn/cm^2^ for 5 min, +8 dyn/cm^2^ for 5 min and then +13 dyn/cm^2^ for the remainder of the experiment. For atheroprone flow, HUVEC were exposed to a repeated cycle of 2 h of oscillatory ±4 dyn/cm^2^ (0.5 Hz), followed by 5 min of unidirectional +4 dyn/cm^2^, to ensure redistribution of nutrients. The ibidi pump apparatus and slide was housed inside a cell culture incubator at 37 °C and 5% CO_2_. The orbital shaker system, as previously described in,^15^ involves orbiting HUVEC using an orbital shaking platform (PSU-10i; Grant Instruments). The radius of the orbital shaker was 10 mm and the rotation rate was set to 210 rpm. HUVEC were grown to confluence on gelatin-coated 6-well plates and then orbited. The rotations of the plate create a shear stress profile where a pulsatile, unidirectional flow pattern of ∼ +13 dyn/cm^2^ is created in the periphery of the well, whereas a tangential flow pattern of ∼ +4 dyn/cm^2^ is generated in the centre of the well. The orbital shaker platform was enclosed inside a cell culture incubator at 37 °C and 5% CO_2_. In experiments where inflammatory gene expression was assessed following P2X7 or P2X4 antagonism, 10 μM A438079 (Abcam), 10 μM PSB-12062 or DMSO was added, respectively, before flow was applied and re-supplemented 24 h before the end of the experiment. In experiments examining p38 phosphorylation, HUVEC were cultured under flow for 72 h using the orbital shaker system before application of 300 μM 2'(3')-O-(4-Benzoylbenzoyl) adenosine-5'-triphosphate (BzATP) under static conditions. For examination of P2X7 involvement in p38 phosphorylation under flow, HUVEC were cultured under flow using the orbital shaker system for 72 h, where for the last 30 min of flow, HUVEC were incubated with 10 μM A438079.

### 2.4 Calcium imaging

HUVEC were loaded with 5 μM CAL-520 (Stratech), diluted in HUVEC cell culture media from a 5 mM stock in anhydrous DMSO, with 1: 1000 pluronic F-127. After a 90-min incubation, HUVEC were washed twice with extracellular imaging buffer (134.3 mM NaCl, 5 mM KCl, 1.2 mM MgCl_2_, 1.5 mM CaCl_2_, 10 mM HEPES, 8 mM Glucose, sterile-filtered, pH 7.4). Calcium imaging was performed by epifluorescence microscopy using a Nikon Eclipse T*i.* Slides were clipped onto the microscope stage at 37 °C and 300 μM BzATP was flushed through the slides. When appropriate, CaCl_2_ was replaced in the extracellular imaging buffer with 0.4 mM ethylene glycol-bis(β-aminoetyhl ether)- N, N, N', N'-tetraacetic acid (EGTA). In experiments where ER calcium stores were depleted, 10 μM thapsigargin was added 3 min before BzATP stimulation, since stores were previously assessed as being depleted by this time point (data not shown). 10 μM A438079 hydrochloride, 10 μM PSB-12062 and 100 µM ARL67156 (Tocris) were incubated with cells in extracellular imaging buffer for 5 min before (or just prior to, for ARL67156) BzATP stimulation. Calcium responses were normalized to the maximal peak of atheroprone flow to generate a percentage maximum response per donor, which was performed to account for donor variability. For dose response experiments, calcium responses from static HUVEC were measured using a BMG labtech FLUOstar OPTIMA plate reader.

### 2.5 Western blotting

HUVEC were lysed directly in laemlli buffer (2% (w/v) SDS, 5% (v/v) β-mercaptoethanol, 10% (v/v) glycerol in 60 mM Tris-HCL, pH 6.8) and boiled at 95 °C for 5 min. Protein was then resolved on a 4-12% bis-tris gel (Invitrogen) in MES buffer at 200 V for 35 min. Proteins were then transferred onto a PVDF membrane at 35 V for 1 h at room temperature. After blocking for 1 h in 5% (w/v) milk in tris buffered saline [1% (v/v) Tween] (TBS-T) or 5% (w/v) BSA TBS-T, membranes were incubated with primary antibodies overnight at 4 °C. Blots were washed three times in TBS-T then incubated for 1 h with HRP-conjugated secondary antibodies. Membranes were washed three more times in TBS-T then visualized by chemiluminescence using ECL-select (GE-Healthcare). Chemiluminescence was detected using a LiCOR c-digit blot scanner and densitometry was determined using Image Studio (LiCOR Biosciences). Band intensities were normalized against PDHX. Data were analysed as densitometry to PDHX but presented as fold change.

### 2.6 qRT-PCR

RNA was isolated using the isolate II RNA mini kit (Bioline) and reverse transcribed to cDNA using iScript cDNA synthesis kit (BioRad). Relative gene expression was then measured by quantitative real time PCR (qRT-PCR) using gene specific primers. iTaq universal SYBR green supermix (Biorad) and corresponding manufacturer’s instructions were used to perform qRT-PCR. Each reaction was performed in triplicate, with the Ct value averaged. Ct values were normalized using the Ct value of Hypoxanthine-guanine phosphoribosyltransferase (HPRT) to generate ΔCt values. Statistics were performed on ΔCt values, but fold changes are shown, calculated using the ΔΔCt method. Gene gene specific primer sequences used for qPCR were:

HPRT Fwd: TTGGTCAGGCAGTATAATCC; Rev: GGGCATATCCTACAACAAAC

IL-8 Fwd: GGCACAAACTTTCAGAGACAG, Rev: ACACAGAGCTGCAGAAATCAGG

E-selectin Fwd: GCTCTGCAGCTCGGACAT Rev: GAAAGTCCAGCTACCAAGGGAAT

### 2.7 Enzyme-linked immunosorbent assay 

Commercial enzyme-linked immunosorbent assay (ELISA) kits (R&D systems) were used to detect IL-8 in cell culture supernatant using the corresponding manufacturer’s instructions. Standards and samples were run in duplicate and optical density absorbance was measured at 450 nm using an absorbance plate reader (Thermo Scientific Varioskan Flash). Concentrations of samples were determined by interpolation from a four-parameter logistic standard calibration curve. Data were analysed as pg/mL but presented as fold change.

### 2.8 Flow cytometry

After flow conditioning, disassociated HUVECs were incubated with CD39-FITC (Clone A1, Biolegend) and Zombie UV viability dye (Biolegend) in Ca^2+^/Mg^2+^ free phosphate buffered saline [0.25% (v/v) foetal bovine serum] for 40 min at 4 °C. Cells were then washed twice by re-suspension. Fluorescence was measured using a LSRII flow cytometer (BD Bioscience), with the median fluorescent intensity measured on live cells.

### 2.9 Gene silencing

HUVEC were transfected via electroporation using the Neon transfection system (Invitrogen; 1200 V, 40 ms, 1 pulse), where expression of P2X7 was silenced using SMARTpool: ON-TARGETplus P2RX7 siRNA (siP2X7, 50 nM) (Dhamacon, L-003728-00-0005) and compared with control non-targeting siRNA (siNC, 50 nM) (Dharmacon, LD001810-10-20). HUVEC were seeded into complete growth medium in 6-well plates, allowed to settle for 2 h then cultured under flow using the orbital shaker system for 72 h. Knockdown was assessed by qPCR and immunoblotting as above, where experiments showing >50% efficiency were compared.

### 2.10 ATP detection assay

HUVEC were cultured under flow using the ibidi pump system for 72 h and extracellular ATP was detected in HUVEC supernatant using a commercially available luciferase-based ATP determination assay (Thermo Fisher).

### 2.11 Mouse lines and *en face* immunostaining

The animal experiments were performed according to the guidelines from Directive 2010/63/EU of the European Parliament on the protection of animals used for scientific purposes and the UK Scientific Procedures Act 1986 (ASPA, licence number 70/7992), under local ethical approval. C57BL/6 mice were used to examine P2X7 expression and BALB/c mice (wild-type or P2X7^−^^/^^−^^16^) were used to examine E-selectin expression. Six- to eight-week old female wild type mice were sacrificed by anaesthetic overdose with pentobarbital (i.p. 200 mg/kg). Exsanguination was performed via cardiac puncture before the aorta was perfused *in situ* with PBS before perfusion-fixation with 4% (v/v) paraformaldehyde (PFA) before harvesting. Ribcage segments, including the aorta, were further fixed for 1 h in 2% (v/v) PFA at room temperature. Aortae were dissected and blocked and permeabilized in PBS (20% (v/v) goat serum, 0.5% (v/v) triton X-100) overnight at 4 °C. Primary antibodies were incubated in PBS-T [5% (w/v) BSA, 0.1% (v/v) tween 20] overnight at 4 °C. Aortae segments were washed three times in PBS before incubation with appropriate AlexaFluor conjugated secondary antibodies in PBS-T at room temperature for 5 h. After a further three washes in PBS, aortae were stained with TO-PRO3 for 1 h at room temperature. Samples were washed three more times in PBS before being mounted. Fully stained vessels were visualized using confocal laser-scanning microscopy (Zeiss LSM510 NLO inverted microscope), where endothelial cells were identified by strong positive CD31 immunostaining. IgG isotype controls (Invitrogen) were run to determine antibody specificity. Three fields-of-view were taken from the atheroprotected and atheroprone site in each mouse. Images were analysed using Image J software, where the expression of the protein of interest was quantified by measuring fluorescence intensity and averaging the measurements from the three fields of view. Fluorescent measurements from the IgG control were subtracted from the staining, as this represented a non-specific or autofluorescent component. Cell surface quantification was predicted by creating a mask from the cell surface CD31 staining and measuring only the fluorescent intensity of the protein of the region within this mask. It should be noted that CD31 can undergo endocytosis and rapid recycling under some circumstances, such as diapedesis.^17^

### 2.12 Statistics

Differences between samples were analysed using a paired Students *t*-test or two-way ANOVA with the Sidak multiple comparison test, as appropriate (*P < 0.05, **P < 0.01, ***P < 0.001, ****P < 0.0001). All analyses were performed on un-normalized data.

## 3. Results

### 3.1 Atheroprone flow primes endothelial cells for ATP-dependent calcium influx

**Figure 1:**
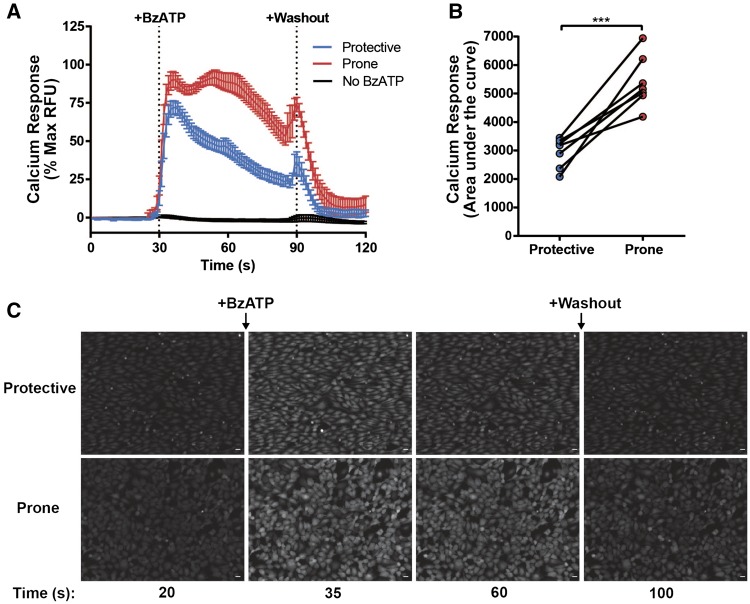
HUVECs pre-conditioned under atheroprone flow exhibit an enhanced ATP-induced calcium response. BzATP (300 µM)-induced calcium response in HUVEC pre-conditioned with atheroprotective or atheroprone flow for 72 h (*A*) and analysed by measuring the area under the curve (*B*) (*n* = 7, *** indicates *P* ≤ 0.001 using a paired *t*-test.). (*C*) Representative screenshots display the change in calcium response over time before and after BzATP treatment (scale=50µm). Values are mean ± S.E.M.

Live cell calcium imaging was used to measure endothelial cell responses to ATP. Calcium responses were identified in HUVEC in response to a range of ATP and BzATP (2′(3′)-*O*-(4-Benzoylbenzoyl)adenosine-5′-triphosphate triethylammonium salt) doses (see Supplementary material online, *Figure S1A and B*). 300 µM BzATP produced a sustained calcium elevation which was attenuated in the absence of extracellular calcium (see Supplementary material online, *Figure S1B*). HUVEC were conditioned under either atheroprotective (+13 dyn/cm^2^) or atheroprone (±4 dyn/cm^2^) flow for 72 h and then stimulated with exogenous BzATP, which exibits higher potency at P2X7 receptors than ATP, a broadly acting agonist at P2X and Purinergic receptors Y (P2Y). Atheroprone flow-conditioned HUVEC exhibited an enhanced and more sustained calcium response to BzATP than those pre-conditioned under atheroprotective flow (*Figure **1**A* and *C*). The total calcium response was measured by analysing the area under the curve, which was significantly increased after atheroprone flow conditioning (*Figure **1**B*). As a control, it was shown that the calcium response occurred specifically in response to the addition of BzATP, as no response was seen when extracellular buffer alone was applied to the cells (*Figure **1**A*). These data indicate that atheroprone flow primes endothelial cells for an enhanced ATP response.

Regulation of extracellular ATP has already been reported to be altered by shear stress, with endothelial expression of the cell surface ATPase CD39 up-regulated by atheroprotective flow.^18^ Therefore, CD39 was examined as a potential mechanism regulating the difference in BzATP-induced calcium responses between atheroprotective and atheroprone flow conditions. Matching previous reports,^18^ CD39 surface expression in HUVEC was significantly enhanced under atheroprotective flow conditions (see Supplementary material online, *Figure S2A*). Furthermore, extracellular levels of ATP were significantly higher in the supernatant of HUVEC cultured under atheroprone flow than atheroprotective flow (see Supplementary material online, *Figure S2B*). In order to determine if enhanced CD39 expression regulated the BzATP-induced calcium response, a chemical inhibitor of CD39, ARL-67156, was used. Inhibition of CD39 evoked an enhanced BzATP-induced calcium responses in HUVEC conditioned under atheroprotective flow (see Supplementary material online, *Figure S2C*), but not under atheroprone flow (see Supplementary material online, *Figure S2D*). Therefore, this suggests that enhanced CD39 activity contributes to reducing ATP signalling under atheroprotective flow.

We tested whether enhanced responses to BzATP were also due to changes in either P2Y or P2X receptor activity. BzATP has been reported to activate all P2X receptors and a sub-set of P2Y receptors.^19^^-^^22^ P2X receptors are ion channels, so are responsible for calcium influx from the extracellular space, whereas P2Y receptors are G-protein coupled receptors and mobilize intracellular calcium stores in response to ATP (similar to other endothelial stimuli including thrombin, histamine and bradykinin whose activation mobilizes intracellular calcium). Therefore, stimulating HUVEC with ATP whilst blocking either extracellular or stored intracellular calcium allows discrimination between P2X and P2Y receptor responses.

**Figure 2:**
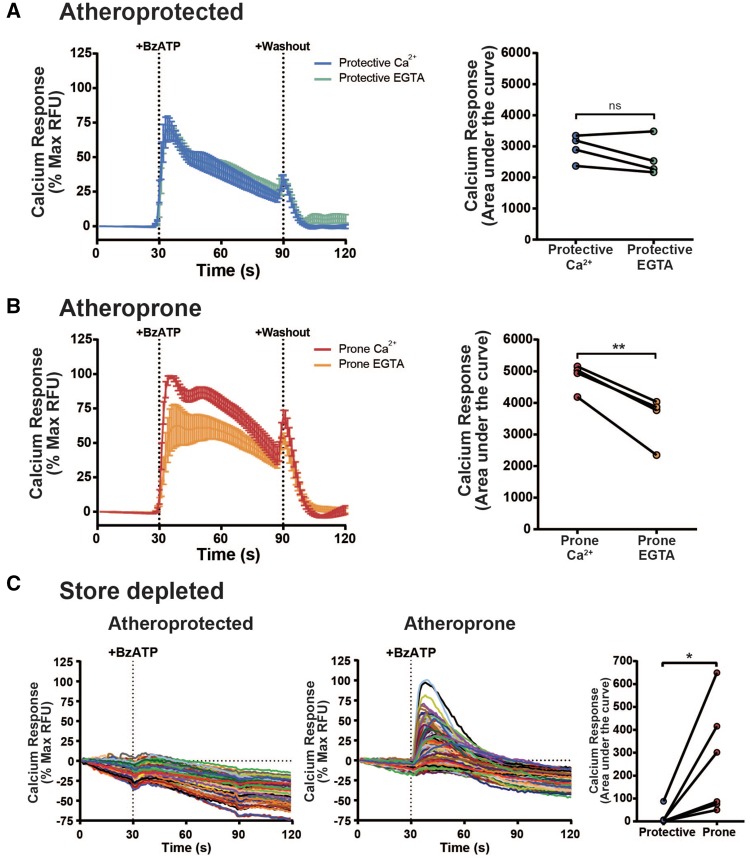
Extracellular calcium influx in response to ATP is increased under atheroprone flow. (*A*) BzATP (300 µM)-induced calcium responses in HUVEC pre-conditioned with atheroprotective flow in the presence of physiological extracellular calcium or EGTA, analysed by measuring area under the curve (*n* = 4). (*B*) BzATP (300 µM) induced calcium responses in HUVEC pre-conditioned with atheroprone flow in the presence of physiological extracellular calcium or EGTA, analysed by measuring area under the curve (*n* = 4, ** indicates *P* ≤ 0.01 using a paired *t*-test). (*C*) Representative single cell BzATP-induced calcium responses in atheroprone or atheroprotective flow conditioned HUVEC after 10 µM thapsigargin pre-treatment, analysed by measuring the average area under the curve (right-hand graph, *n* = 6, 175 cells per donor, *indicates *P* ≤ 0.05 using a paired *t*-test). Values are mean ± S.E.M.

Initially, extracellular calcium was substituted with the calcium chelator EGTA, to prevent BzATP-mediated calcium influx, whilst maintaining release from intracellular calcium stores. In HUVEC pre-conditioned with atheroprotective flow, there was no significant difference in the calcium response between calcium- or EGTA-containing extracellular buffer (*Figure **2**A*), indicating an absence of extracellular calcium influx. In contrast, HUVEC pre-conditioned with atheroprone flow showed a reduction in the calcium response in the presence of EGTA (*Figure **2**B*), indicating a significant contribution from extracellular calcium influx.

To substantiate this finding, the converse experiment was performed where extracellular calcium influx was maintained and intracellular calcium mobilization was inhibited. To achieve this, intracellular ER calcium stores were depleted using thapsigargin prior to BzATP stimulation. Depletion of ER calcium stores led to a significant reduction in BzATP-dependent calcium influx in HUVEC exposed to atheroprotective flow compared with BzATP-mediated influx responses under atheroprone flow (*Figure **2**C*). Together these data suggest that extracellular calcium influx in response to ATP occurs exclusively under atheroprone flow. Since P2X ion channels mediate calcium influx in response to ATP, this suggests that endothelial P2X receptors are selectively activated under atheroprone flow.

### 3.2 Expression of ATP-gated P2X7 ion channels was enhanced under atheroprone flow

**Figure 3:**
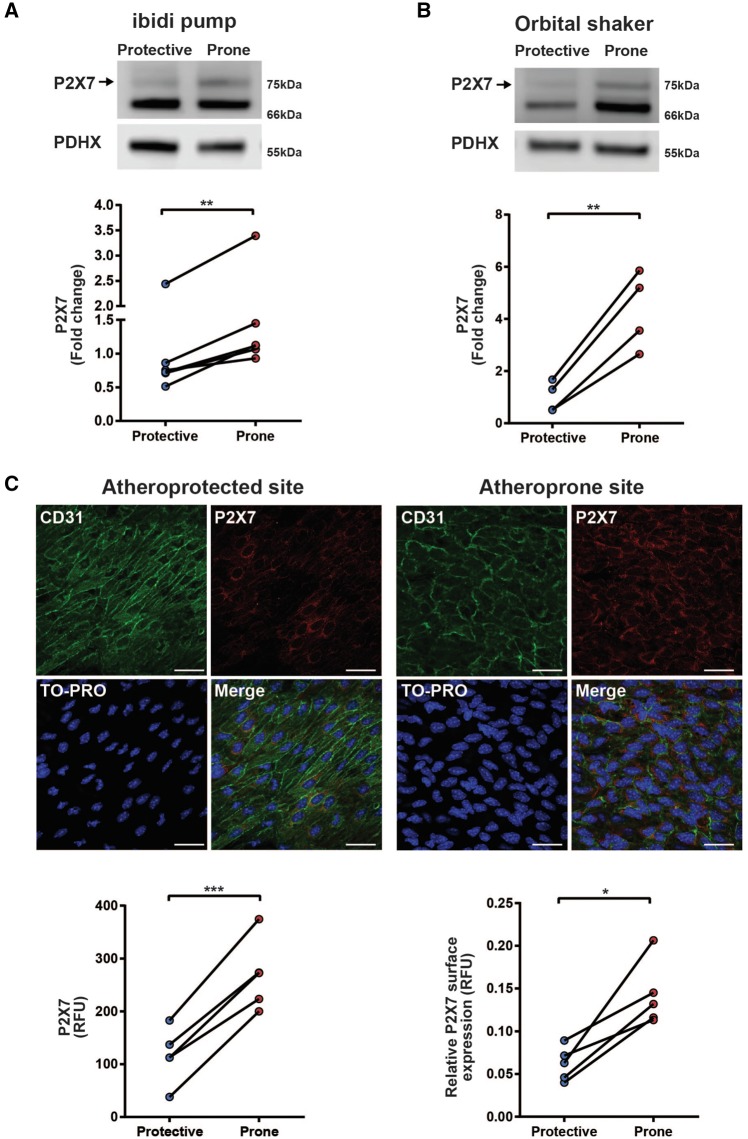
Expression of ATP-gated P2X7 receptors is enhanced under atheroprone flow. (*A*) Western blot and densitometry of P2X7 in flow conditioned HUVEC using the ibidi flow system (*n* = 6, ** indicates *P* ≤ 0.01 using a paired *t*-test). P2X7 is shown by an arrow, validated by P2X7 knockdown, shown in Supplementary material online, *Figure *4B. (*B*) Western blot and densitometry of P2X7 in flow conditioned HUVEC using the orbital shaker system (*n* = 4, ** indicates *P* ≤ 0.01 using a paired *t*-test). (*C*) Representative *en face* immunostaining for P2X7 (red) on wildtype C57BL/6 mice at atheroprotective (outer curvature) and atheroprone (inner curvature) regions of the aorta (scale = 25 µm). Endothelial cells were identified by staining with CD31 (green) and cell nuclei were stained using TO-PRO (blue). P2X7 expression was analysed by measuring the relative fluorescent intensity at sites protected or prone to atherosclerosis. Surface expression was measured by measuring P2X7 fluorescence at sites co-localized with the endothelial cell surface marker CD31. Relative fluorescent intensities for P2X7 were corrected against the relative fluorescent intensity of IgG performed on the descending aorta (*n* = 5, * indicates *P* ≤ 0.05 and *** indicates *P* ≤ 0.001 using a paired *t*-test). Values are mean ± S.E.M.

Since atheroprone flow-conditioned HUVEC exhibited a selective increase in extracellular calcium influx, P2X receptor involvement was assessed. Previous studies have shown that P2X4 and P2X7 receptors are more abundantly expressed in endothelial cells compared with other P2X receptors.^22^^,^^23^ We found that expression of P2X7 was increased in HUVEC cultured under atheroprone flow in two complementary *in vitro* flow models (*Figure **3**A* and *B*). Expression of P2X4 was also induced by atheroprone flow but to a modest extent compared with P2X7 (see Supplementary material online, *Figure S3A*).

To examine if P2X7 receptors were also regulated *in vivo* by shear stress, expression of endothelial P2X7 at sites protected and prone to atherosclerosis in the murine aorta was examined using *en face* immunostaining. Specific regions of the mouse aorta have been mapped by computation fluid dynamics where the outer curvature, exposed to high shear stress, is considered an atheroprotected site, and the inner curvature, exposed to disturbed flow is an atheroprone site.^24^ In agreement with our *in vitro* findings using HUVEC, endothelial P2X7 expression was increased at sites prone to atherosclerosis compared with protected sites (*Figure **3**C*). Analysing P2X7 co-localization with the cell surface endothelial marker CD31 also showed a significant increase at sites of atheroprone flow compared with atheroprotected regions, indicating that surface P2X7 levels may be increased (*Figure **3**C*). Analysis of the CD31/P2X7 co-staining also allowed for adjustment in differences between relative cell membrane proportions since the elongated aligned morphology in the atheroprotected region confers an increase in cell membrane, despite a small reduction in density. As a control, P2X7 antibodies were shown to interact specifically to P2X7 by *en face* immunostaining of endothelial cells in the descending aorta of P2X7^−^^/^^−^ mice (see Supplementary material online, *Figure S4*). These data support our observations that an up-regulation of P2X7 receptors occurs due to chronic exposure to disturbed blood flow.

### 3.3 P2X7 receptors are required for enhanced inflammatory signalling under atheroprone flow

**Figure 4:**
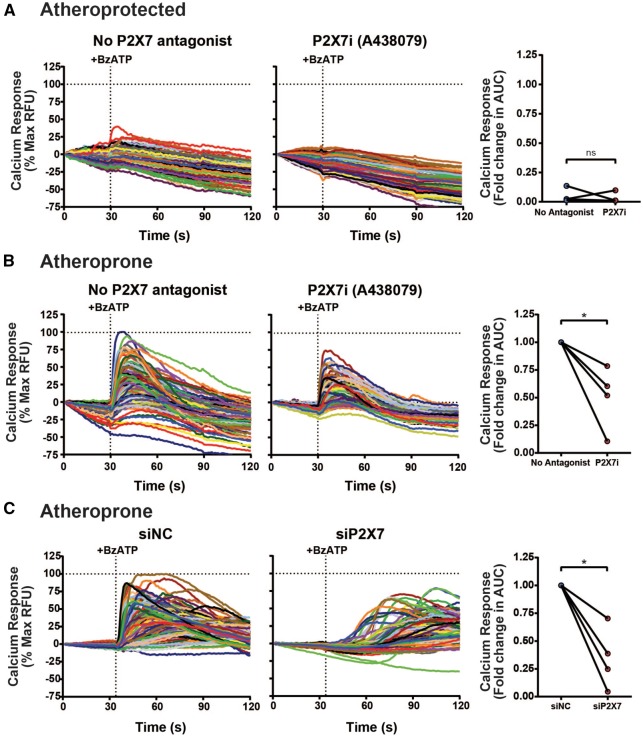
P2X7 receptor activity occurs exclusively in HUVEC under atheroprone flow. Representative single cell ATP-induced calcium responses in atheroprotected (*A*) or atheroprone (*B, C*) flow conditioned HUVEC after thapsigargin pre-treatment ± the P2X7 inhibitor 10 µM A438079 (P2X7i) or siRNA control and P2X7 knockdown (*C*) and analysed by measuring the average area under the curve (*n* = 4, 175 cells per donor, * indicates *P* ≤ 0.05 using a paired *t*-test). Values are mean ± S.E.M.

The function of endothelial P2X receptors was assessed using antagonists. Application of the P2X7 antagonist A438079 hydrochloride resulted in a ∼50% reduction in the extracellular calcium influx response to ATP under atheroprone flow (*Figure **4**B*); calcium influx was negligible in atheroprotected cells in the absence and presence of this antagonist (*Figure **4**A*). Knockdown of P2X7 using siRNA also reduced BzATP-induced calcium influx in atheroprone flow conditioned HUVEC (*Figure **4**C*). Conversely, P2X4 inhibition in atheroprone flow conditioned HUVEC using PSB-12062 did not alter extracellular calcium influx (see Supplementary material online, *Figure S3B*). It was concluded that atheroprone flow enhanced calcium signalling, via an up-regulation of P2X7 receptor expression.

**Figure 5:**
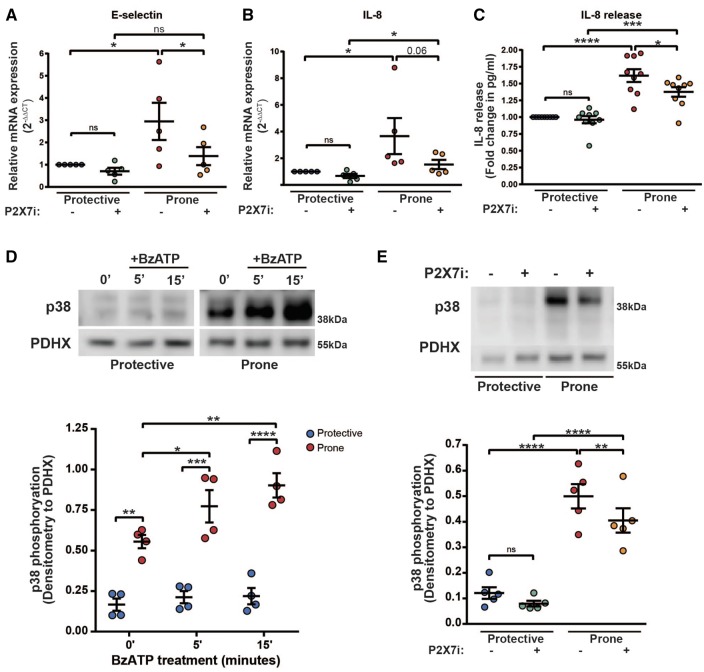
Atheroprone flow mediated inflammatory signalling is regulated by P2X7 responses. qPCR analysis of E-selectin (*A*) and IL-8 (*B*) in HUVEC conditioned with atheroprotective or atheroprone flow using the ibidi system ± the P2X7 inhibitor 10 µM A438079 (P2X7i) (*n* = 5, * indicates *P* ≤ 0.05 using a two-way ANOVA). (*C*) IL-8 release measured by ELISA in the supernatant of HUVEC conditioned under atheroprotective or atheroprone flow using the ibidi system ± the P2X7 inhibitor 10 µM A438079 (P2X7i) (*n* = 9, * indicates *P* ≤ 0.05, *** indicates p = <0.001 and **** indicates *P* ≤ 0.0001 using a two-way ANOVA). (*D*) Representative western blot and densitometry for p38 phosphorylation following 5- or 15-min treatment of BzATP (300 µM) in atheroprotective or atheroprone flow conditioned HUVEC (*n* = 4, * indicates *P* ≤ 0.05, ** indicates *P* ≤ 0.01, *** indicates *P* ≤ 0.001 and **** indicates *P* ≤ 0.0001 using a two-way ANOVA). (*E*) Representative western blot and densitometry of p38 phosphorylation in atheroprone or atheroprotective flow conditioned HUVEC using the orbital shaker system ± 30-min treatment with the P2X7 inhibitor 10 µM A438079 (P2X7i) (*n* = 5, ** indicates *P* ≤ 0.01, **** indicates *P* ≤ 0.0001 using a two-way ANOVA). Values are mean ± S.E.M.

Next we studied the effects of enhanced P2X7 activity on inflammatory signalling under atheroprone flow. Atheroprone flow significantly enhanced expression of IL-8 and E-selectin (*Figure **5**A* and *B*). P2X7 receptor inhibition reduced induction of E-selectin and IL-8, with both declining to similar levels of those expressed by HUVEC under atheroprotective flow (*Figure **5**A* and *B*). Secretion of IL-8, determined by ELISA of the cell culture supernatant, was also increased under atheroprone flow compared with atheroprotective flow and was significantly reduced by P2X7 inhibition specifically under atheroprone flow (*Figure **5**C*). These effects were specific to P2X7, as P2X4 inhibition with PSB-12062 did not alter expression of IL-8 or E-selectin (see Supplementary material online, *Figure S3C–E*). eNOS expression was unaffected by P2X7 inhibition (data not shown), while NFATc1 (but not other NFAT transcripts) was downregulated in endothelial cells exposed to atheroprone flow and this expression was further attenuated in the presence of the P2X7 antagonist (data not shown). This suggests that the increased P2X7 responsiveness observed in the atheroprone exposed cells is unlikely coupled to NFAT activation or to eNOS expression. Overall, these data suggest that endothelial P2X7 receptors are activated by endogenously produced ATP under atheroprone flow and are required for the induction of inflammatory signalling.

In order to identify the signalling mechanisms leading to E-selectin and IL-8 up-regulation, p38 phosphorylation in response to ATP was examined. p38 has been reported to be activated by atheroprone flow^25^ and by P2X7,^26^ but these mechanisms have not been linked in endothelial cells. p38 was phosphorylated rapidly following BzATP stimulation in atheroprone, but not atheroprotected, flow conditioned HUVEC (*Figure **5**D*) suggesting that ATP signalling is sufficient to activate p38 signalling in these cells. Next, P2X7 receptor antagonists were used to identify if P2X7 mediates p38 phosphorylation under atheroprone flow conditions. In agreement with previous reports, p38 phosphorylation was significantly increased under atheroprone flow compared with atheroprotective flow (*Figure **5**E*). Interestingly, inhibition of P2X7 significantly reduced p38 phosphorylation under atheroprone flow conditions (*Figure **5**E*). These data indicate that the induction of inflammatory molecules by atheroprone flow is mediated via a P2X7-p38 signalling pathway.

**Figure 6:**
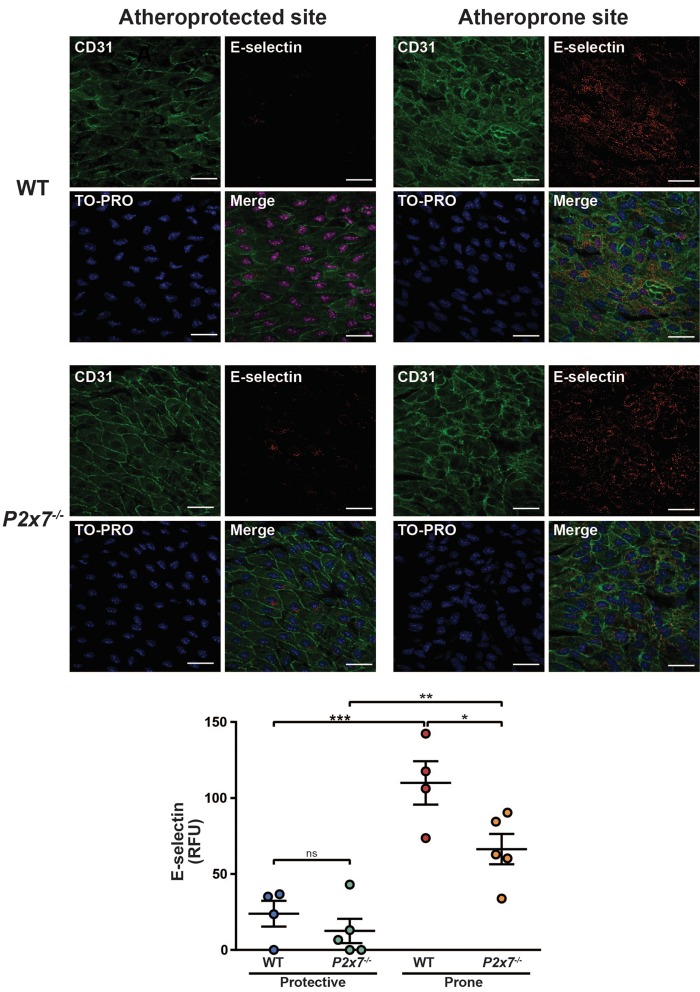
P2X7 regulates E-selectin expression at sites susceptible to atherosclerosis *in vivo*. Representative *en face* immunostaining for E-selectin (red) on wildtype or P2X7^−/−^ BALB/c mice at atheroprotective (outer curvature) and atheroprone (inner curvature) sites of the aorta (*n* = 4–5) (scale = 25 µm). Endothelial cells were identified by staining with CD31 (green) and cell nuclei were stained using TO-PRO (blue). E-selectin expression was analysed by measuring the relative fluorescent intensity at sites of protected or prone to atherosclerosis (*n* = 4–5, * indicates *P* ≤ 0.05, ** indicates *P* ≤ 0.01 and *** indicates *P* ≤ 0.001 using a two-way ANOVA). Relative fluorescent intensities for P2X7 were corrected against the relative fluorescent intensity of IgG. Values are mean ± S.E.M.

To examine if P2X7 regulates atheroprone flow-mediated inflammatory signalling *in vivo*, *en face* immunostaining for E-selectin at atheroprotected and atherosusceptible sites of the murine aorta was performed using wild-type and P2X7^−^^/^^−^ mice. E-selectin expression was significantly enhanced at sites predisposed to atherosclerosis (*Figure **6*). In agreement with our *in vitro* data using HUVEC (derived from a non-arterial source), we found that expression of E-selectin at atheroprone sites was significantly reduced in P2X7^−^^/^^−^ mice compared with wild-type, suggesting that P2X7 regulates endothelial inflammation *in vivo* at sites prone to atherosclerosis development.

In summary, our data reveal that atheroprone flow enhances inflammation via an ATP-dependent P2X7-p38 signalling pathway.

## 4. Discussion

Mechanical shear stress influences the focal nature of atherosclerosis by activating multiple signalling pathways that influence endothelial cell function. At bends and branch points of arteries disturbed flow generates shear stress with low magnitude and oscillations that promotes inflammatory activation. Here we show for the first time that atheroprone flow primes endothelial cells for enhanced purinergic signalling which drives vascular inflammation via a calcium-p38 signalling pathway. The underlying mechanism relies on low shear stress-dependent up-regulation of P2X7 at the cell surface, which responds to ATP by mediating calcium influx. The atheroprone shear stress up-regulation of P2X7 receptor expression was observed at the protein level, and also at the cell surface at atherosusceptible sites when measured *in vivo*. We concluded that shear stress regulation of P2X7 is downstream from transcription or mRNA stability because P2X7 mRNA levels were not enhanced by atheroprone flow (data not shown). Interestingly P2X7 receptors are associated with caveolae in osteoblasts,^27^ cardiomyocytes,^28^ and alveolar epithelial cells,^29^^,^^30^ while post-translational modifications direct P2X7 to lipid microdomains.^31^ Thus we speculate that caveolae, which are shear sensitive,^32^ may play a role in atheroprone-mediated up-regulation and clustering of P2X7 at the cell surface thereby enhancing calcium influx and inflammatory signalling in response to ATP.

Our observation that atheroprone flow sensitizes endothelial cells for responses to ATP complements previous studies showing that disturbed flow patterns can modulate local ATP levels through its degradation, synthesis and release. Of note, *in vivo* studies revealed that the ATPase CD39 is highly expressed under atheroprotected high shear stress exposure, thereby rapidly hydrolyzing ATP at the cell surface.^18^ In contrast CD39 is down-regulated in atherosusceptible regions of the murine aorta and CD39 deficient mice on an Apolipoprotein E (ApoE)^−^^/^^−^ background exhibited increased atherosclerosis.^18^ Similarly we observed significantly reduced CD39 expression in atheroprone compared with atheroprotected endothelial cells in our *in vitro* models, and an enhancement of the BzATP mediated calcium response in atheroprotected cells following CD39 inhibition. In addition to reduced hydrolysis, ATP levels are also actively increased in response to atheroprone flow and inflammation by: augmented cell surface F_1_/F_0_ ATP synthase activity within caveolae^33^^,^^34^; increased activation of pannexins^35^; and release of ATP-rich vesicles.^36^^,^^37^ Together these studies reveal that ATP generation and hydrolysis are regulated by shear stress, leading to enhanced ATP levels at atheroprone sites. Collectively, our observations and those from previous reports indicate that endothelial cells at atheroprone sites display enhanced ATP-dependent signalling via two complementary mechanisms; accumulation of ATP and sensitization to ATP via P2X7 receptor up-regulation.

Although purinergic signalling has been linked to multiple diverse endothelial functions its contribution to focal atherogenesis was uncertain. Here we demonstrate that enhanced P2X7-dependent ATP signalling promotes endothelial inflammatory activation at atheroprone sites via a calcium-dependent signalling pathway. This observation is consistent with previous studies demonstrating that extracellular ATP can induce expression of adhesion molecules^7^^,^^10^^,^^38^^,^^39^ and chemokines^10^^,^^40^^,^^41^ and promote leukocyte adhesion to the endothelium.^7–10^ Moreover recent studies have shown that administration of extracellular ATP to ApoE^−^^/^^−^ mice exacerbated atherosclerosis by increasing leukocyte adhesion and migration.^9^ Furthermore, inflammatory stimuli such as LPS^42^, TNF,^35^ hypoxia,^36^ and high glucose/palmitate^10^ all increase extracellular ATP release from endothelial cells, further supporting a role of extracellular ATP in endothelial inflammatory responses. Thus we suggest that mechanical shear-dependent upregulation of P2X7 at atheroprone sites may be important in the initiation of atherosclerosis by transducing extracellular ATP into pro-inflammatory signalling responses. Our studies of the molecular mechanism underlying P2X7-dependent inflammatory activation identified a potential role for p38 MAP kinase. Although P2X7 has not been linked previously to p38 activation in atheroprone endothelium, P2X7 activation is known to promote p38 phosphorylation in other cell types.^3^^,^^43^^,^^44^ Our observations also support considerable evidence demonstrating a role for p38 activity in vascular inflammation,^45^^,^^46^ atherosclerosis,^25^^,^^47–49^ and specifically in IL-8 and E-selectin activation.^50–52^

Our findings align with previous studies linking P2X7 with atherosclerosis. Recently a role for P2X7 in lesion development, atherosclerosis, and plaque inflammation was demonstrated using cholesterol fed P2X7^−^^/^^−^LDLR^−^^/^^−^ mice.^14^ P2X7 was found to drive inflammasome activation in plaque macrophages. Interestingly leukocyte rolling and adhesion was reduced in P2X7 deficient mice,^14^ linking directly to our discovery that P2X7 activation promotes E-selectin upregulation at endothelial sites exposed to atheroprone flow. This recent work by Stachon *et al*. (2017)^14^ did not detect P2X7 staining in CD31 positive endothelial cells, although cross-sectional histology was performed and expression at atheroprone arterial regions was not explored, whilst our study addressed this question specifically using the more sensitive *en face* staining technique, showing P2X7 expression in atheroprone endothelium which was considerably lower at atheroprotective arterial sites. Our findings are also consistent with clinical epidemiological evidence that a loss-of-function polymorphism in the human P2X7 receptor has been associated with a reduced risk of ischaemic heart disease and ischaemic stroke.^53^ Our study provides novel insight, demonstrating a role for P2X7 in mediating endothelial inflammation following chronic exposure to atheroprone flow, an early event in atherosclerosis development. Thus P2X7 likely plays a role in both focal initiation of lesion formation as well as plaque progression. P2X7 has already been extensively studied as a potential therapeutic target in inflammation. As a result, several P2X7 receptor antagonists are available,^54^^,^^55^ for use in pre-clinical and clinical studies.^56^ Future studies should therefore address which P2X7 inhibitors can prevent the initiation of atherosclerotic lesions by dampening local inflammation.

In summary, endothelial P2X7 is essential for atheroprone flow-induced inflammatory signalling *in vitro* and at atheroprone sites *in vivo.* Since P2X7 is activated specifically at atheroprone sites linked to dysregulation of localized extracellular ATP levels, we propose its activity contributes to early endothelial dysfunction preceding atherosclerosis development.

## Supplementary material

Supplementary material is available at *Cardiovascular Research* online.

## Supplementary Material

Supplementary Figures

Supplementary Figure Legends
