# Supplementary material for: Atheroprone flow activates inflammation via endothelial ATP-dependent P2X7-p38 signalling
Source: Cardiovasc Res. 2017 Nov 6;114(2):324–35. doi: 10.1093/cvr/cvx213 (PMC5852506; doi:10.1093/cvr/cvx213)

Sup Figure 1

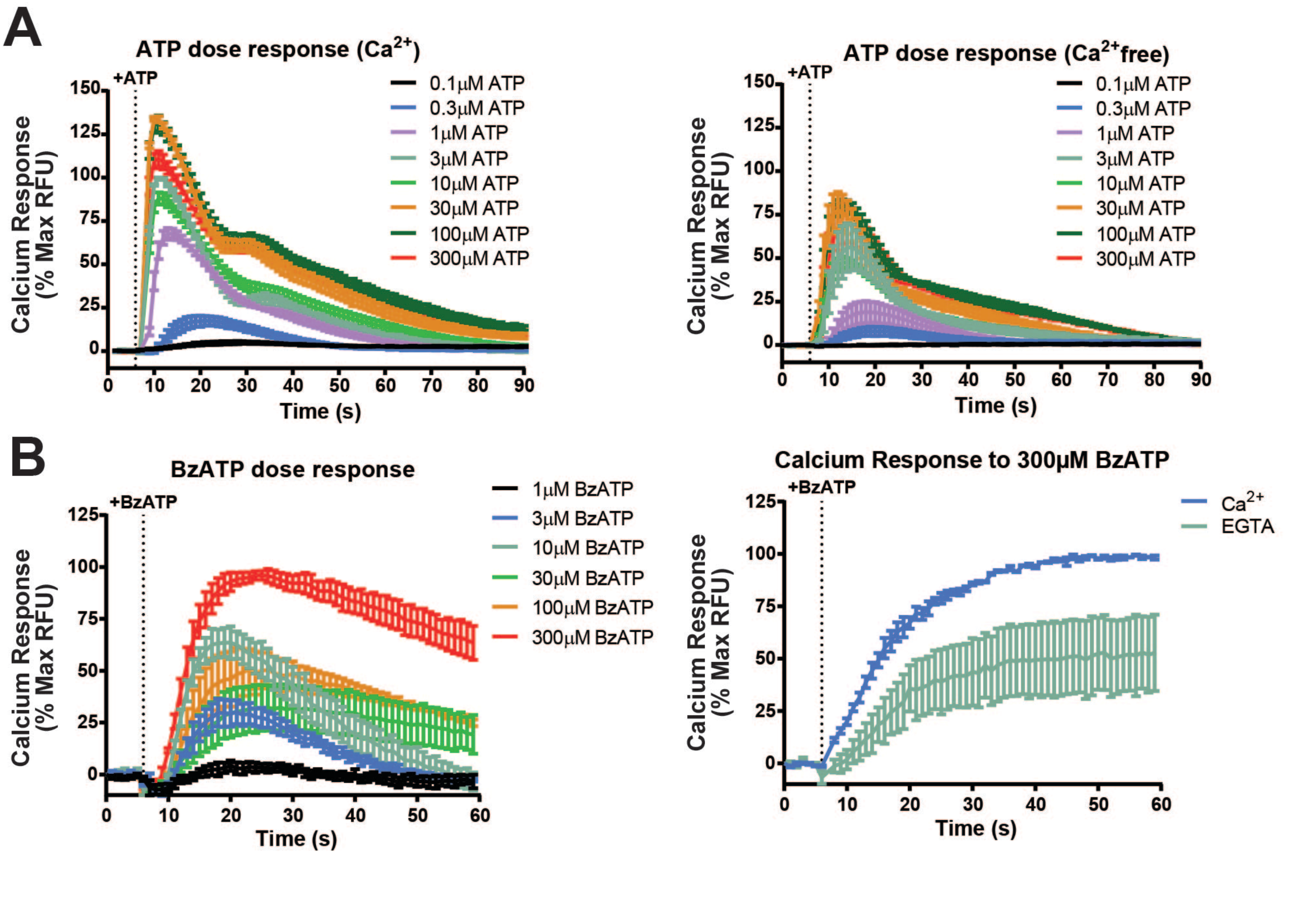

A
ATP dose response (Ca2+)
+ATP
Calcium Response (% Max RFU)
Time (s)
0.1μM ATP
0.3μM ATP
1μM ATP
3μM ATP
10μM ATP
30μM ATP
100μM ATP
300μM ATP
ATP dose response (Ca2+free)
B
BzATP dose response
+BzATP
1μM BzATP
3μM BzATP
10μM BzATP
30μM BzATP
100μM BzATP
300μM BzATP
Calcium Response to 300μM BzATP
Ca2+
EGTA

Sup Figure 2

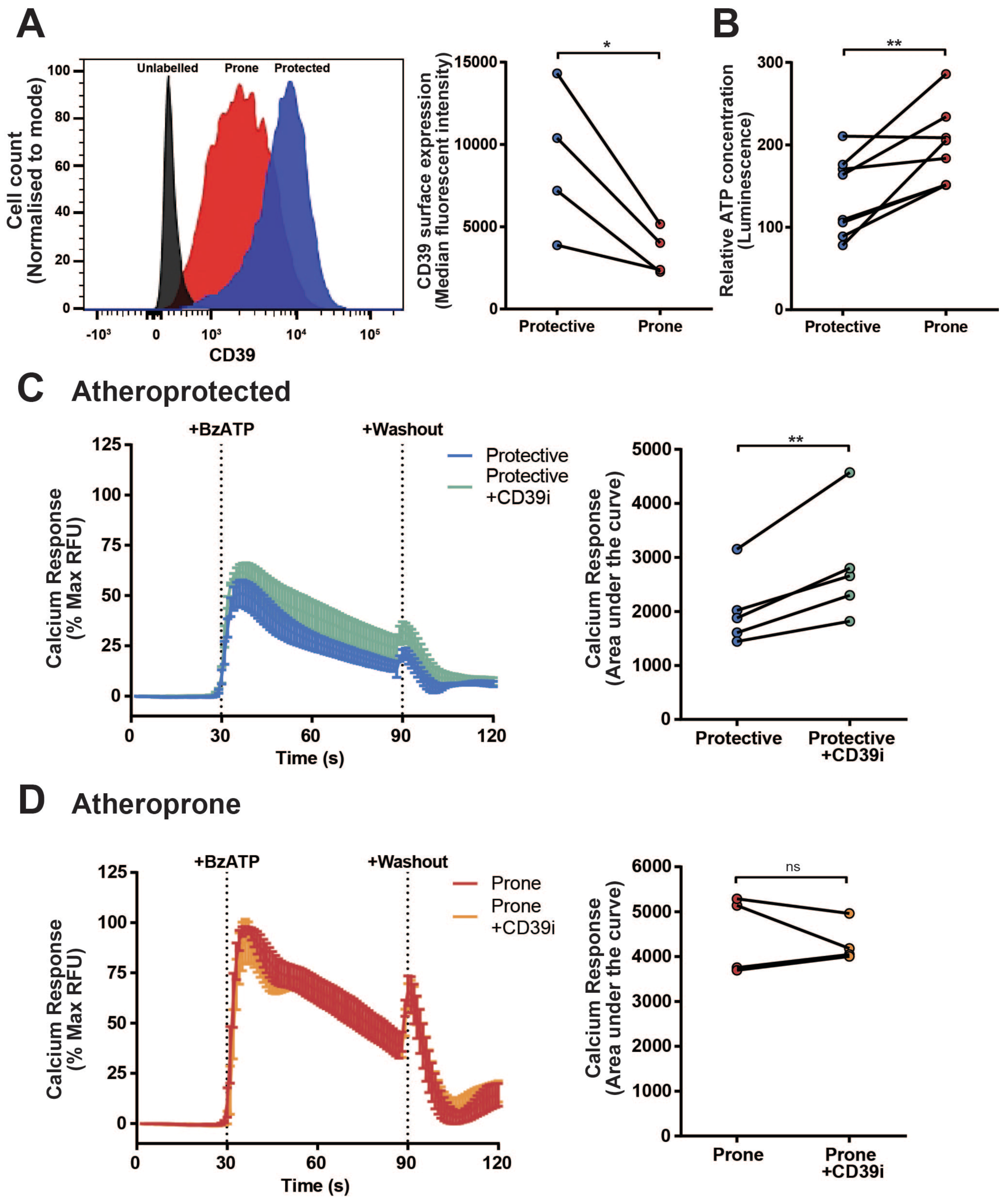
A
Cell count
(Normalised to mode)
100
80
60
40
20
0
Unlabelled
Prone
Protected
-10³
0
10³
10⁴
10⁵
CD39
CD39 surface expression
(Median fluorescent intensity)
15000
10000
5000
0
*
Protective
Prone
B
Relative ATP concentration
(Luminescence)
300
200
100
0
**
Protective
Prone
C
Atheroprotected
+BzATP
+Washout
Protective
Protective
+CD39i
Calcium Response
(% Max RFU)
125
100
75
50
25
0
0
30
60
90
120
Time (s)
Calcium Response
(Area under the curve)
5000
4000
3000
2000
1000
0
**
Protective
Protective
+CD39i
D
Atheroprone
+BzATP
+Washout
Prone
Prone
+CD39i
Calcium Response
(% Max RFU)
125
100
75
50
25
0
0
30
60
90
120
Time (s)
Calcium Response
(Area under the curve)
6000
5000
4000
3000
2000
1000
0
ns
Prone
Prone
+CD39i

Sup Figure 3

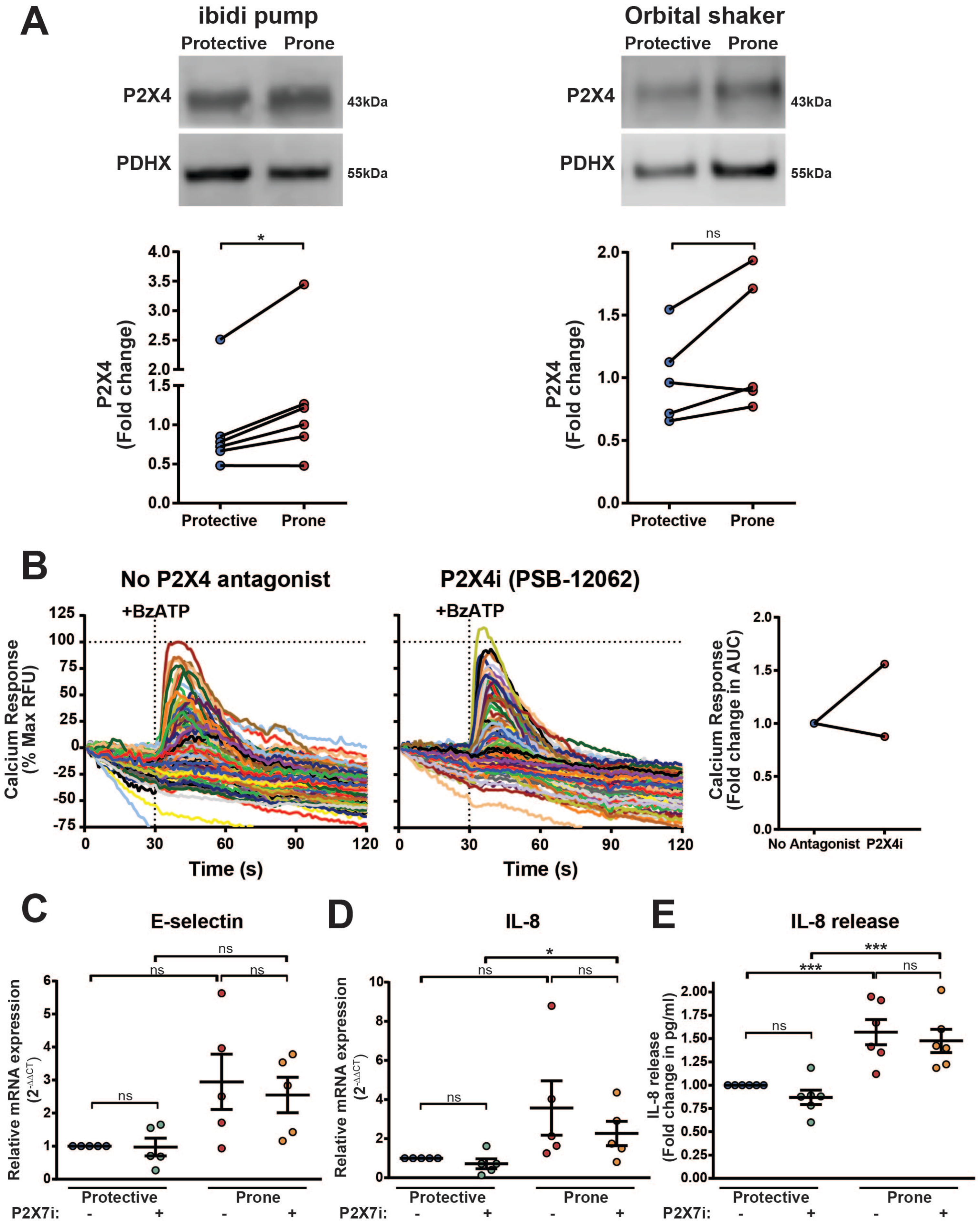

A
ibidi pump
Protective
Prone
P2X4
43kDa
PDHX
55kDa
Orbital shaker
Protective
Prone
P2X4
43kDa
PDHX
55kDa
*
P2X4 (Fold change)
4.0
3.5
3.0
2.5
2.0
1.5
1.0
0.5
0.0
Protective
Prone
ns
P2X4 (Fold change)
2.0
1.5
1.0
0.5
0.0
Protective
Prone
B
No P2X4 antagonist
+BzATP
Calcium Response (% Max RFU)
125
100
75
50
25
0
-25
-50
-75
0
30
60
90
120
Time (s)
P2X4i (PSB-12062)
+BzATP
0
30
60
90
120
Time (s)
Calcium Response (Fold change in AUC)
2.0
1.5
1.0
0.5
0.0
No Antagonist
P2X4i
C
E-selectin
Relative mRNA expression ($2^{-\Delta\Delta CT}$)
6
5
4
3
2
1
0
ns
ns
ns
ns
Protective
Prone
P2X7i:
-
+
-
+
D
IL-8
Relative mRNA expression ($2^{-\Delta\Delta CT}$)
10
8
6
4
2
0
*
ns
ns
ns
Protective
Prone
P2X7i:
-
+
-
+
E
IL-8 release
IL-8 release (Fold change in pg/ml)
2.00
1.75
1.50
1.25
1.00
0.75
0.50
0.25
0.00
***
***
ns
ns
Protective
Prone
P2X7i:
-
+
-
+

Sup Figure 4

A

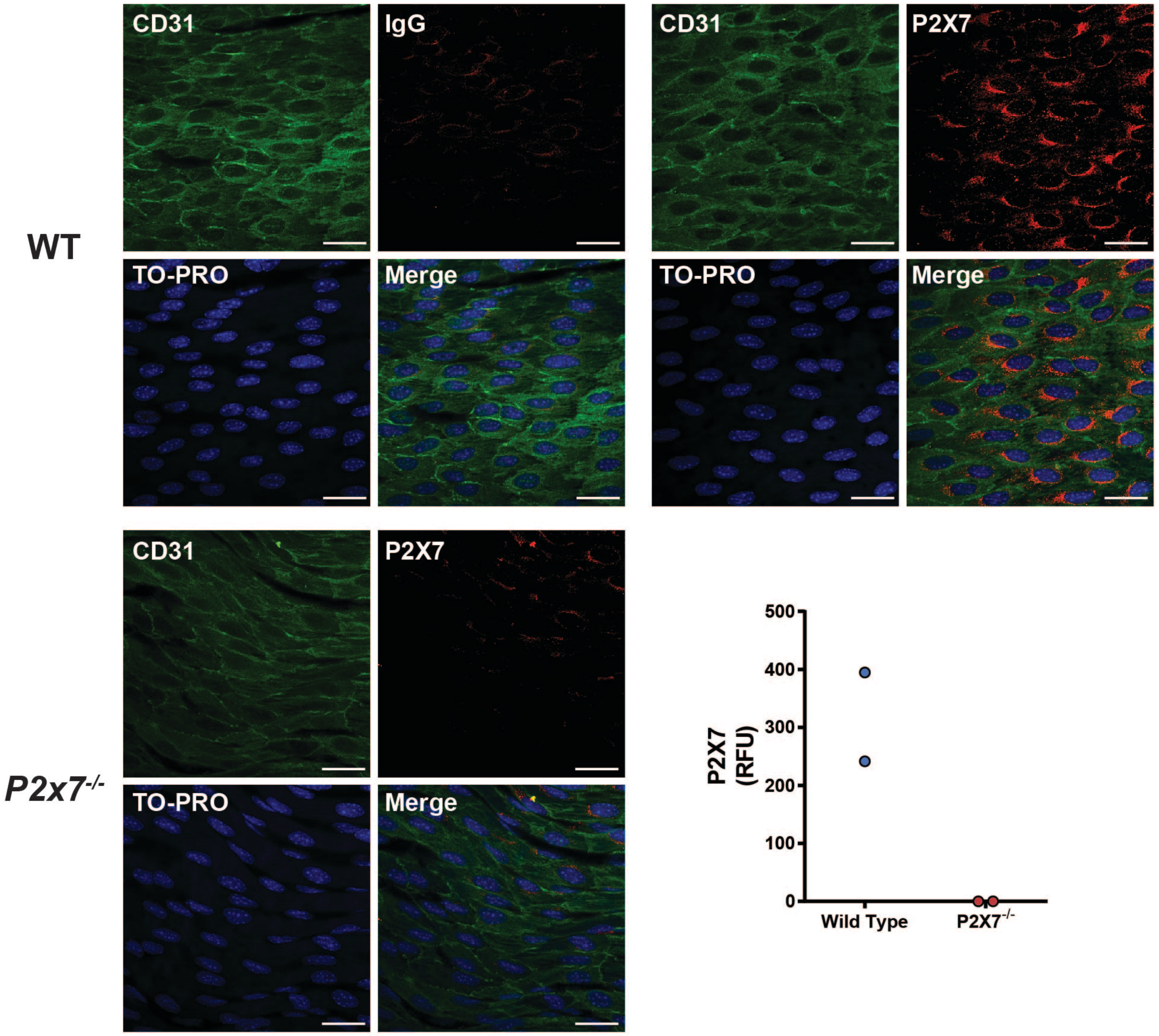

B

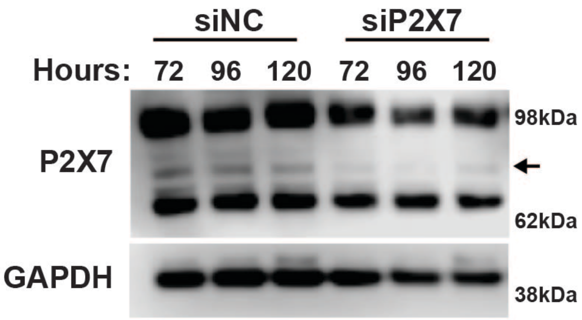

Supplement: Supplementary Figures [file supfigures_cvx213.pdf]
