# Supplementary material for: Atheroprone flow activates inflammation via endothelial ATP-dependent P2X7-p38 signalling
Source: Cardiovasc Res. 2017 Nov 6;114(2):324–35. doi: 10.1093/cvr/cvx213 (PMC5852506; doi:10.1093/cvr/cvx213)
Supplement: Supplementary Figure Legends [file supplementary_figure_legends_cvx213.docx]

**Figure S1: Dose response curves for ATP and BzATP in HUVEC.**

(A) Calcium responses in static HUVEC to increasing doses of ATP in the presence (left) or absence (right) of extracellular Ca^2+^. (B) Calcium responses in static HUVEC to increasing doses of BzATP in the presence of extracellular Ca^2+^ (left). BzATP (300µM) induced calcium response in static HUVEC ± 400µM EGTA to chelate extracellular Ca^2+^ (right). Values are mean ± SEM or as means paired to individual donors.

**Figure S2: Endothelial CD39 expression regulates ATP signalling under atheroprotective flow.**

(A) Surface CD39 expression on atheroprotective (blue) or atheroprone (red) flow conditioned HUVEC measured by flow cytometry (n=4, * indicates p=<0.05 using a paired *t*-test). (B) Determination of ATP levels in supernatants of atheroprotective or atheroprone flow conditioned HUVEC using a luciferase based luminescence assay (n=8, ** indicates p=<0.01 using a paired *t­*-test). (C) BzATP (300µM) induced calcium responses in HUVEC preconditioned with atheroprotective flow ± the CD39 inhibitor ARL67156 (CD39i, 100µM) and analysed by measuring the average area under the curve (n=5, ** indicates p=<0.01 using a paired *t*-test). (D) BzATP (300µM) induced calcium responses in HUVEC preconditioned with atheroprone flow ± the CD39 inhibitor ARL67156 (CD39i, 100µM) and analysed by measuring the average area under the curve (n=4, ns, not significantly different using a paired *t*-test). Values are mean ± SEM or as means paired to individual donors.

**Figure S3: Assessment of P2X4 function in atheroprone conditioned HUVEC.**

(A) Western blot and densitometry of P2X4 in deglycosylated (PNGase-F treated) lysate from flow conditioned HUVEC using the ibidi flow system (n=6, * indicates p=<0.05 using a paired *t*-test) or the orbital shaker system (n=4). (B) Representative single cell BzATP (300µM) induced calcium responses in atheroprone conditioned HUVEC after thapsigargin pre-treatment (10µM, 3 minutes) ± a P2X4 inhibitor (PSB-12062, 10µM) and analysed by measuring the average area under the curve per (n=2, 175 cells per donor). qPCR analysis of E-selectin (C) and IL-8 (D) in atheroprone or atheroprotective flow conditioned HUVEC ± P2X4i (PSB-12062, 10µM) (n=5). (E) ELISA measured IL-8 release in the supernatant of HUVEC conditioned under atheroprone flow ± P2X4i (PSB-12062, 10µM) (n=6). * indicates p=<0.05 and *** indicates p=<0.001 using a two way ANOVA. Values are mean ± SEM or as means paired to individual donors.

**Figure S4: P2X7 antibodies are specific for endothelial *en face* immunostaining and in western blotting**

(A) Representative *en face* immunostaining for P2X7 or IgG (red) in the descending aorta of wildtype or P2x7^-/-^ BALB/c mice. Endothelial cells were identified by strong CD31 (green) immunostaining and nuclei (blue) were stained with TO-PRO. Expression of P2X7 was determined by assessing relative fluorescence intensity corrected against the IgG control (n=2). (B) Western blot for P2X7 in HUVEC treated with control non-targeting (siNC) siRNA or siRNA targeting P2X7 (siP2X7) transcripts for the indicated time. The arrow indicates the band (~75kDa) most sensitive to P2X7 silencing. Values shown are mean.
